# Age comorbidity scores as risk factors for 90‐day mortality in patients with a pancreatic head adenocarcinoma receiving a pancreaticoduodenectomy: A National Population‐Based Study

**DOI:** 10.1002/cam4.2730

**Published:** 2019-12-02

**Authors:** Ben‐Chang Shia, Lei Qin, Kuan‐Chou Lin, Chih‐Yuan Fang, Lo‐Lin Tsai, Yi‐Wei Kao, Szu‐Yuan Wu

**Affiliations:** ^1^ Research Center of Big Data College of management Taipei Medical University Taipei Taiwan; ^2^ College of Management Taipei Medical University Taipei Taiwan; ^3^ Executive Master Program of Business Administration in Biotechnology College of Management Taipei Medical University Taipei Taiwan; ^4^ School of Statistics University of International Business and Economics Beijing China; ^5^ Division of Oral and Maxillofacial Surgery Department of Dentistry Wan Fang Hospital Taipei Medical University Taipei Taiwan; ^6^ School of Dentistry College of Oral Medicine Taipei Medical University Taipei Taiwan; ^7^ Graduate Institute of Business Administration Fu Jen Catholic University Taipei Taiwan; ^8^ Department of Food Nutrition and Health Biotechnology College of Medical and Health Science Asia University Taichung Taiwan; ^9^ Division of Radiation Oncology Lo‐Hsu Medical Foundation Lotung Poh‐Ai Hospital Yilan Taiwan; ^10^ Big Data Center Lo‐Hsu Medical Foundation Lotung Poh‐Ai Hospital Yilan Taiwan; ^11^ Department of Healthcare Administration College of Medical and Health Science Asia University Taichung Taiwan; ^12^ Department of Radiology School of Medicine College of Medicine Taipei Medical University Taipei Taiwan

**Keywords:** 90‐day mortality, comorbidity, older age, pancreatic head adenocarcinoma, pancreaticoduodenectomy

## Abstract

**Background:**

To estimate easily assessed preoperative factors for predicting 90‐day mortality in patients with a pancreatic head adenocarcinoma (PHA) receiving a pancreaticoduodenectomy.

**Methods:**

We analyzed data from the Taiwan Cancer Registry Database of patients with a PHA who received a pancreaticoduodenectomy. Basic demographic characteristics, including gender and age, were categorized. The selection of preoperative comorbidities was based on the preoperative American Society of Anesthesiologists score and Charlson comorbidity index.

**Results:**

We enrolled 8490 patients with a PHA who received a pancreaticoduodenectomy without distant metastasis. Currently, a pancreaticoduodenectomy for a PHA achieves an overall 90‐day mortality rate of 8.39%. Univariate and multivariate Cox regression analyses indicated that an older age (65‐74 and ≥75 years) and specific comorbidities (chronic obstructive pulmonary disease, chronic kidney disease, dementia, and sepsis) were significant independent prognostic factors for predicting 90‐day mortality after a pancreaticoduodenectomy. After adjustment, the adjusted hazard ratios (aHRs) (95% confidence intervals [CIs]) of subjects with middle and high comorbidity scores for 90‐day mortality in 65 to 74‐year‐old patients were 1.36 (1.05‐1.75) and 2.25 (1.03‐4.90), respectively, compared to subjects with low comorbidity scores. The aHRs (95% CIs) of subjects with middle and high comorbidity scores for 90‐day mortality in ≥75‐year‐old patients were 1.35 (1.07‐1.78) and 2.07 (1.19‐3.62), respectively, compared to those with low comorbidity scores.

**Conclusions:**

Elderly patients with a PHA and moderate or high comorbidity scores have an increased risk of 90‐day mortality after a pancreaticoduodenectomy.

## Graphical abstract

This is the first study to utilize the largest cohort of patients with pancreatic head adenocarcinoma and to develop an easily implementable tool using preoperative characteristics such as age‐comorbidity predictive scores for 90‐day mortality in patients with pancreatic head adenocarcinoma receiving pancreaticoduodenectomy.

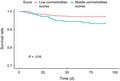

## 1 INTRODUCTION

Pancreatic adenocarcinomas (PAs) are a highly lethal malignancy.^1^, ^2^ They are the fourth leading cause of cancer‐related deaths in the United States and second only to colorectal cancer as a cause of digestive cancer‐related deaths.^2^ In Taiwan, PAs are the fifth leading cause of cancer‐related deaths in women and the eighth leading cause of cancer‐related deaths in men.^3^, ^4^, ^5^ Surgical resection is the only potentially curative treatment for PA.^6^ The most common anatomic location of a PA is the head (approximately 70%).^7^ The standard operation for a pancreatic head adenocarcinoma (PHA) is a pancreaticoduodenectomy.^8^, ^9^

A pancreaticoduodenectomy is a complex operation, and its early mortality rate is high.^10^, ^11^, ^12^ One of the most important reasons for improvements in pancreaticoduodenectomies is the considerable experience of a limited number of surgeons who perform the operation on a regular basis in high‐volume hepatobiliary centers.^13^, ^14^, ^15^ However, the definitions of experience and learning curves differ depending on individual surgeons’ learning ability and continuing medical education.^16^, ^17^, ^18^ Cutoff values in high‐volume hospitals are vague and different^13^, ^19^, ^20^ and vary with different surgical procedures.^18^, ^19^, ^21^ Easily assessed tools for predicting early perioperative mortality with a pancreaticoduodenectomy are needed as a reference for pancreaticoduodenectomies or other alternative treatments for patients with a PHA.

All events recorded within 90 days of resection are considered postoperative complications.^22^ Instead of 30‐day mortality, 90‐day postoperative mortality is a valid measure of hepatopancreatobiliary surgical quality.^23^ Moreover, 30‐day mortality usually underestimates the risk of early death after major resections for malignancies.^10^, ^24^, ^25^ Therefore, in this study, we attempted to estimate easily assessed preoperative factors for predicting 90‐day mortality in patients with a PHA who underwent a pancreaticoduodenectomy. These novel easily assessed preoperative factors may represent an easy and useful tool for evaluating 90‐day mortality to prevent underestimating perioperative mortality if using the 30‐day mortality rate after a pancreaticoduodenectomy for patients with a PHA.

## 2 PATIENTS AND METHODS

### 2.1 Data source

The Taiwan Cancer Registry Database (TCRD) from the Collaboration Center of Health Information application contains detailed cancer‐related information,^5^, ^26^, ^27^, ^28^, ^29^, ^30^, ^31^, ^32^, ^33^, ^34^ and the quality and precision of codes in Taiwan were verified and proven by previous studies.^35^, ^36^

### 2.2 Study cohort

We established a cohort from the TCRD. We enrolled patients who had received a diagnosis of resectable PHA and then undergone a pancreaticoduodenectomy between January 1, 2006 and December 31, 2015. The index date was the date of surgery. The follow‐up duration was from the index date to December 31, 2016. Our protocols were reviewed and approved by the Institutional Review Board of Taipei Medical University. The diagnoses of enrolled patients were confirmed according to their pathological data, and patients who received a new diagnosis of resectable PHA and underwent pancreaticoduodenectomy surgery exhibited no other cancer or distant metastasis. The inclusion criteria were a diagnosis of resectable PHA with the indication for a pancreaticoduodenectomy, being aged ≥20 years, having an adenocarcinoma, and being at clinical cancer stage I to III without metastasis as per the American Joint Committee on Cancer (AJCC) Cancer Staging Manual, Seventh Edition. Induction chemotherapy was used in our study. The exclusion criteria were a history of cancer before the pancreatic head cancer diagnosis, missing gender data, unclear staging, neoadjuvant radiotherapy before the index date, and non‐adenocarcinoma histology.

### 2.3 Study covariates

Basic demographic characteristics, including gender and age, were categorized. Patient age was determined according to the index date; patients were accordingly divided into five age groups: <45, 45‐54, 55‐64, 65‐74, and ≥75 years. The variables of interest included demographic characteristics, AJCC clinical stage, comorbidities, hospital volume,^19^, ^37^ and income level.^38^ Comorbidities were derived on the basis of previous studies and the Taiwan National Health Insurance Research Database (TCRD).^39^, ^40^, ^41^ The selection of comorbidities was based on the preoperative American Society of Anesthesiologists score and Charlson comorbidity index. Patients with diabetes mellitus (DM), hypertension (HTN), pneumonia, chronic obstructive pulmonary disease (COPD), hepatitis B, hepatitis C, acute myocardial infarction (AMI), coronary artery disease (CAD), cerebrovascular disease (CVD), heart valve dysfunction, sepsis, chronic kidney disease (CKD), heart failure, aortic aneurysm, peripheral vascular disease, peptic ulcer disease, dementia, chronic pulmonary disease, connective tissue disease, mild liver disease, hemiplegia, moderate or severe renal disease, and moderate or severe liver disease were examined. To ensure relevance, comorbidities reported >1 year before the index date were not included. Based on the main International Classification of Diseases, Ninth Revision, Clinical Modification (ICD‐9‐CM) diagnostic codes, comorbidities were identified with a positive diagnosis in a single admission or a positive diagnosis in two or more visits to outpatient departments within 1 year before the index date.

### 2.4 Endpoint

The endpoint was the 90‐day mortality rate among patients who received a pancreaticoduodenectomy.

### 2.5 Statistical analysis

A Chi‐squared test was used to compare demographic characteristics between surviving patients and the 90‐day mortality rate between those with and without a specific comorbidity (Table 1). Independent predictors were adjusted for and stratified in the analysis. Univariate and multivariate Cox proportional hazard models were constructed. Significant independent predictors—such as old age and specific significant comorbidities—were analyzed using multivariate Cox regression models, which were also used to calculate hazard ratios (HRs). After adjusting for confounders, Cox proportional hazards were used to model the time from the index date to 90‐day mortality for enrolled patients. In the multivariate analysis, HRs were adjusted for age, gender, the aforementioned comorbidities, clinical AJCC stage, hospital volume, and income level (Table 1). Table 3 presents stratified analyses performed to evaluate the 90‐day mortality risk associated with different independent significant comorbidities mentioned in Table 2; the analyses were stratified by low, middle, and high comorbidity scores (0, 1 or 2, and 3 or 4) and different age groups (<45, 45‐64, 65‐74, and ≥75 years). All analyses were performed using SAS (vers. 9.3; SAS). Two‐tailed *P* < .05 was considered statistically significant. The 90‐day survival rate was estimated using the Kaplan‐Meier method. Differences among patients with low, middle, and high comorbidity scores were determined using the log‐rank test.

**Table 1:** 90‐day survival rates stratified by the characteristics of patients with pancreatic head adenocarcinoma receiving pancreaticoduodenectomy

	No. of patients	Survival rate, n (%)		*P* value
	8490	7778	91.61%	
Age (years)				<.001
<45	817	786	96.21%	
45‐54	1410	1343	95.25%	
55‐64	2203	2077	94.28%	
65‐74	2521	2274	90.20%	
≥75	1539	1298	84.34%	
Gender				.107
Female	3491	3219	92.21%	
Male	4999	4559	91.20%	
Comorbidities				
Diabetes mellitus	3086	2775	89.92%	<.001*
Hypertension	4543	4072	89.63%	<.001*
Pneumonia	2879	2601	90.34%	.003*
COPD	2116	1863	88.04%	<.001*
Hepatitis B	684	641	93.71%	.046*
Hepatitis C	333	297	89.19%	.127*
Myocardial infarction	130	110	84.62%	.006*
CAD	2386	2132	89.35%	<.001*
CVD	1335	1169	87.57%	<.001*
Heart valve dysfunction	576	510	88.54%	.007*
Sepsis	775	677	87.35%	<.001*
CKD	1250	1095	87.60%	<.001*
Heart failure	898	780	86.86%	<.001*
Aortic aneurysm	36	31	86.11%	.372*
Peripheral vascular disease	652	570	87.42%	<.001*
Peptic ulcer disease	4888	4448	91.00%	.019*
Dementia	276	229	82.97%	<.001*
Chronic pulmonary disease	2585	2316	89.59%	<.001*
Connective tissue disease	550	498	90.55%	.393*
Mild liver disease	4716	4365	92.56%	.001*
Hemiplegia	1113	974	87.51%	<.001*
Moderate or severe renal disease	514	455	88.52%	.011*
Moderate or severe liver disease	1481	1357	91.63%	<.001*
Clinical AJCC stage				<.001
I	2918	2705	92.70%	
IIA	3141	2878	91.63%	
IIB	2103	1903	90.50%	
III	328	292	89.02%	
Hospital‐volume levels				<.001
Very high	3202	2966	92.63%	
High	3104	2866	92.33%	
Moderate	1525	1353	88.72%	
Low	313	282	90.10%	
Very low	346	311	89.88%	
Income level				<.001
Low	2268	2055	90.61%	
Middle	3907	3543	90.68%	
High	2315	2180	94.17%	

Abbreviations: AJCC, American Joint Committee on Cancer; AMI, acute myocardial infarction; CAD, coronary artery disease; CKD, chronic kidney disease; COPD, chronic obstructive pulmonary disease; CVD, cerebrovascular disease; DM, diabetes mellitus; HTN, hypertension.

* A Chi‐square test was used to compare 90‐day mortality rates between surviving patients with and those without a specific comorbidity.

**Table 2:** Univariate and multivariate Cox proportional hazards analyses of clinical factors for 90‐day mortality in patients with a pancreatic head adenocarcinoma who received a pancreaticoduodenectomy

	Univariate analysis				Multivariate analysis			
	HR	*P* value	95% CI		aHR^a^	*P* value	95% CI	
Age (years)								
<45 (ref.)								
45‐54	1.25	.300	0.82	1.92	1.28	.265	0.83	1.96
55‐64	1.51	.039	1.02	2.24	1.40	.102	0.94	2.08
65‐74	2.65	<.001	1.82	3.84	2.12	<.001	1.44	3.13
≥75	4.36	<.001	3.00	6.34	3.13	<.001	2.11	4.66
Gender								
Female (ref.)								
Male	1.13	.110	0.97	1.32	1.15	.073	0.99	1.34
Comorbidities								
No comorbidity (ref.)								
Diabetes mellitus	1.37	<.001	1.19	1.59	1.13	.126	0.97	1.33
Hypertension	1.74	<.001	1.49	2.03	1.17	.094	0.97	1.40
Pneumonia	1.26	.003	1.08	1.46	1.04	.622	0.89	1.22
COPD	1.70	<.001	1.46	1.98	1.35	.014	1.06	1.71
Hepatitis B	1.73	.044	1.04	1.99	1.76	.124	1.05	1.98
Hepatitis C	1.32	.109	0.94	1.84	1.29	.183	0.89	1.86
Myocardial infarction	1.93	.004	1.24	3.01	1.37	.173	0.87	2.17
CAD	1.44	<.001	1.24	1.68	0.92	.365	0.77	1.10
CVD	1.67	<.001	1.41	1.99	1.05	.754	0.78	1.40
Heart valve dysfunction	1.42	.007	1.10	1.83	1.13	.361	0.87	1.48
Sepsis	1.62	<.001	1.31	2.01	1.40	.003	1.12	1.74
CKD	1.65	<.001	1.38	1.98	1.29	.012	1.06	1.57
Heart failure	1.72	<.001	1.41	2.10	1.10	.391	0.88	1.37
Aortic aneurysm	1.72	.225	0.72	4.16	1.15	.752	0.47	2.82
Peripheral vascular disease	1.60	<.001	1.27	2.02	1.11	.385	0.87	1.42
Peptic ulcer disease	1.20	.018	1.03	1.40	1.05	.564	0.89	1.23
Dementia	2.19	<.001	1.63	2.94	1.42	.027	1.04	1.93
Chronic pulmonary disease	1.40	<.001	1.21	1.63	0.86	.191	0.68	1.08
Connective tissue disease	1.15	.329	0.87	1.53	0.91	.517	0.68	1.21
Mild liver disease	1.77	.049	1.06	1.89	1.73	.487	0.62	1.85
Hemiplegia	1.65	<.001	1.37	1.99	1.02	.908	0.75	1.38
Moderate or severe renal disease	1.43	.009	1.09	1.86	1.03	.850	0.77	1.37
Moderate or severe liver disease	1.00	.990	0.82	1.21	1.13	.305	0.89	1.43
Clinical AJCC stage								
I (ref.)								
IIA	1.19	.670	0.54	2.62	2.12	.176	0.71	6.28
IIB	1.27	.586	0.54	2.99	2.01	.162	0.64	4.08
III	1.31	.950	0.27	3.01	2.32	.956	0.10	4.93
Hospital‐volume level								
Very high (ref.)								
High	1.04	.639	0.87	1.25	1.02	.848	0.85	1.22
Moderate	1.57	.229	0.79	1.91	1.29	.119	0.84	1.60
Low	1.37	.098	0.94	1.99	1.18	.400	0.81	1.72
Very low	1.40	.066	0.98	1.99	1.10	.610	0.76	1.60
Income level								
Low (ref.)								
Middle	1.00	.986	0.84	1.18	1.07	.480	0.89	1.29
High	0.61	.053	0.31	1.01	0.80	.050	0.64	1.00

Abbreviations: aHR, adjusted hazard ratio; AJCC, American Joint Committee on Cancer; AMI, acute myocardial infarction; CAD, coronary artery disease; CI, confidence interval; CKD, chronic kidney disease; COPD, chronic obstructive pulmonary disease; CVD, cerebrovascular disease; DM, diabetes mellitus; HR, hazard ratio; HTN, hypertension.

a All of the variables of Table 1 were used in the multivariate analysis.

## 3 RESULTS

We enrolled 8490 patients with a PHA who underwent a pancreaticoduodenectomy without distant metastasis (Table 1). Of the 8490 enrolled patients, 712 died before completing the 90‐day threshold, whereas 7778 survived; thus, a current pancreaticoduodenectomy for PHA achieved an overall 90‐day mortality rate of 8.39%. High 90‐day mortality rates were observed in patients that received a pancreaticoduodenectomy who were of an old age, had comorbidities, had advanced AJCC stage, had a low income level, or were operated on in a low‐volume hospital (Table 1).

Univariate and multivariate Cox regression analyses indicated that old age (65‐74 and ≥75 years) and specific comorbidities (COPD, CKD, dementia, and sepsis) were crucial independent prognostic factors (Table 2). After the multivariate analysis, being aged 65‐74 and ≥75 years (adjusted HR [aHR]: 2.12; 95% confidence interval [CI]: 1.44‐3.13 and aHR: 3.13; 95% CI: 2.11‐4.66, respectively) were crucial independent prognostic factors for 90‐day mortality. Specific comorbidities such as COPD (aHR: 1.35; 95% CI: 1.06‐1.71), CKD (aHR: 1.29; 95% CI: 1.06‐1.57), dementia (aHR: 1.42; 95% CI: 1.04‐1.93), and sepsis (aHR: 1.40; 95% CI: 1.12‐1.74) were also significant independent prognostic factors for 90‐day mortality (Table 2). Age was determined to be a crucial independent prognostic factor. Furthermore, the aHR increased with an advancement in age from 65‐74 to ≥75 years (aHR: 2.12 and 3.13 for ages 65‐74 and ≥75, respectively; Table 2).

A stratified Cox proportional hazard model assessed the risk of 90‐day mortality and the associated specific comorbidity scores of patients with a resectable PHA who underwent a pancreaticoduodenectomy by considering different age groups (Table 3). We divided the cohort into four age subsets and developed three separate comorbidity scores (low, middle, and high) for patients. A Cox proportional hazard model was used to analyze the 90‐day mortality risk associated with different comorbidity scores at different ages (Table 3). After adjustment, the aHR (95% CIs) of the middle comorbidity score for 90‐day mortality in younger patients (<45 years old) was 1.23 (1.06‐4.32) compared to those with a low comorbidity score. The aHRs (95% CI) of middle and high comorbidity scores for 90‐day mortality in patients aged 45‐64 years were 1.51 (1.13‐2.02) and 2.12 (1.03‐7.13), respectively, compared to those with a low comorbidity score. The aHRs (95% CIs) of middle and high comorbidity scores for 90‐day mortality in patients aged 65‐74 years were 1.36 (1.05‐1.75) and 2.25 (1.03‐4.90), respectively, compared to those with a low comorbidity score. The aHRs (95% CIs) of middle and high comorbidity scores for 90‐day mortality in patients aged ≥75 years were 1.35 (1.07‐1.78) and 2.07 (1.19‐3.62), respectively, compared to those with a low comorbidity score.

**Table 3:** Age‐stratified Cox proportional hazards model for the 90‐days mortality risk associated with specific comorbidities in patients with pancreatic head adenocarcinoma receiving pancreaticoduodenectomy

Age (years)	n	Survival	Death	Death rate (%)	Univariate analysis			Multivariate analysis		
					HR	95% CI	*P* value	aHR^a^	95% CI	*P* value
<45										
Low comorbidity score (0) (Ref.)	636	618	18	2.80						
Middle comorbidity score (1 or 2)	180	168	12	6.67	2.31	1.15‐4.94	.020	1.23	1.06‐4.32	.025
High comorbidity score (3 or 4)	0	0	0	N/A	N/A	N/A	N/A	N/A	N/A	N/A
45‐64										
Low comorbidity score (0) (Ref.)	2513	2398	115	4.60						
Middle comorbidity score (1 or 2)	1075	1000	75	6.98	1.55	1.16‐2.07	.003	1.51	1.13‐2.02	.005
High comorbidity score (3 or 4)	25	22	3	12.00	2.69	0.86‐8.46	.091	2.12	1.03‐7.31	.011
65‐74										
Low comorbidity score (0) (Ref.)	1309	1199	110	8.4						
Middle comorbidity score (1 or 2)	1175	1045	130	11.06	1.33	1.03‐1.72	.027	1.36	1.05‐1.75	.020
High comorbidity score (3 or 4)	37	30	7	18.92	2.40	1.12‐5.16	.024	2.25	1.03‐0.90	.042
≥75										
Low comorbidity score (0) (Ref.)	620	540	80	12.90						
Middle comorbidity score (1 or 2)	859	713	146	17.00	1.35	1.03‐1.77	.032	1.35	1.03‐1.78	.033
High comorbidity score (3 or 4)	60	45	15	25.00	1.97	1.14‐3.43	.016	2.07	1.19‐3.62	.011

Abbreviations: aHR, adjusted hazard ratio; CI, confidence interval; HR, hazard ratio.

a All of the variables in Table 1 were used in the multivariate analysis.

**Figure 1:**
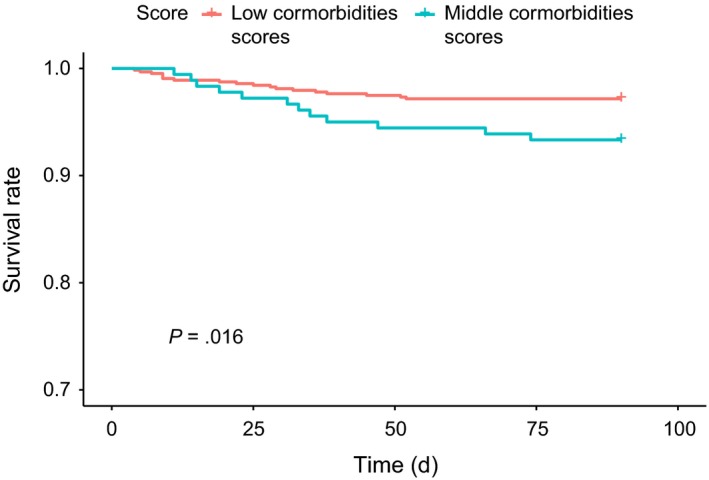
Kaplan‐Meier curves for 90‐day survival after a pancreaticoduodenectomy in <45‐year‐old patients with different comorbidity scores

**Figure 2:**
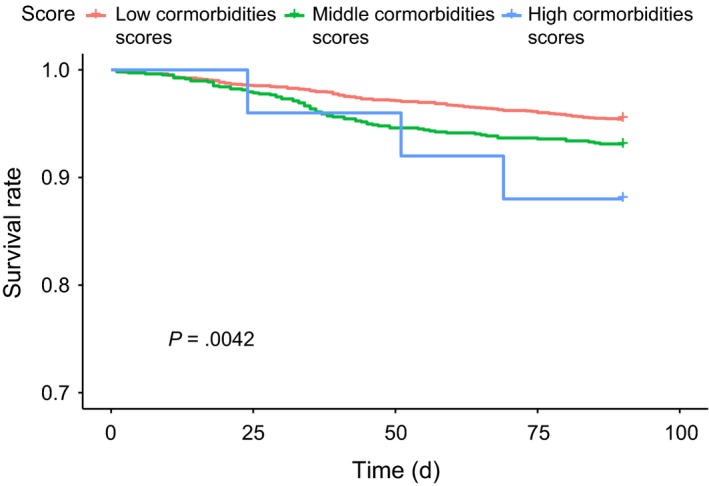
Kaplan‐Meier curves for 90‐day survival after a pancreaticoduodenectomy in 45 to 64‐year‐old patients with different comorbidity scores

**Figure 3:**
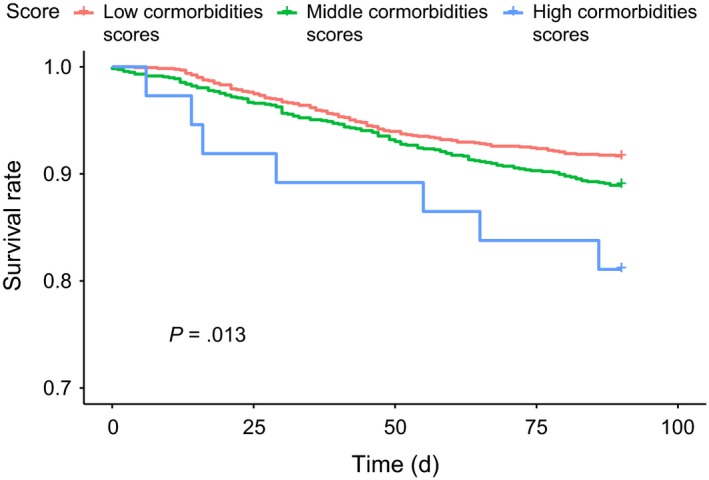
Kaplan‐Meier curves for 90‐day survival after a pancreaticoduodenectomy in 65 to 74‐year‐old patients with different comorbidity scores

**Figure 4:**
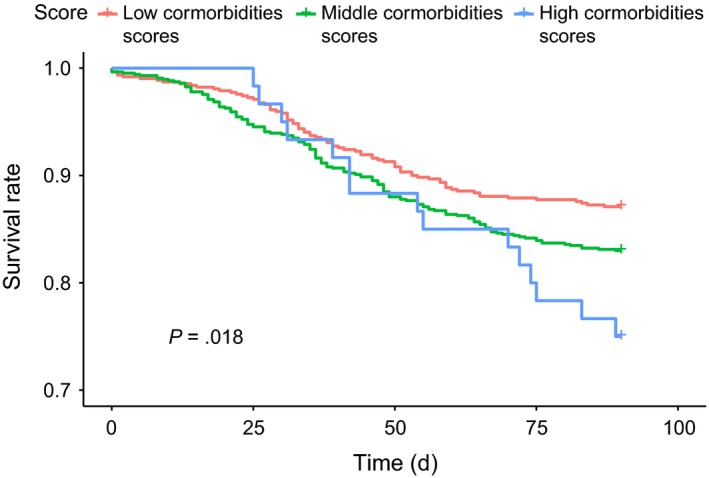
Kaplan‐Meier curves for 90‐day survival after a pancreaticoduodenectomy in ≥75‐year‐old patients with different comorbidity scores

Figure 1 illustrates the Kaplan‐Meier curves of 90‐day survival after a pancreaticoduodenectomy in patients aged <45 year who had different comorbidity scores. The 90‐day survival rates were 93.33% and 97.20% in those with middle and low comorbidity scores, respectively. The 90‐day survival rate was 97.20% in those with low comorbidity scores, which was the highest (log‐rank test, *P* = .016). The 90‐day survival rate in patients aged 45‐64 years who had low (96.40%), middle (93.02%), and high (88.00%) comorbidity scores significantly differed (log‐rank test, *P* = .004, Figure 2). The 90‐day survival rates in elderly patients (65‐74 years old) with low, middle, and high comorbidity scores were 91.60%, 88.94%, and 81.08%, respectively (log‐rank test, *P* = .013, Figure 3). The 90‐day survival rates in elderly patients (≥75 years old) with low, middle, and high comorbidity scores were 87.10%, 83.00%, and 75.00%, respectively (log‐rank test, *P* = .018, Figure 4). Figures 1, 2, 3, 4 indicate that 90‐day survival rates of patients with low comorbidity scores were superior to rates of those with middle and high comorbidity scores across different age groups. For the same comorbidity scores, survival rates were higher in younger patients (Table 3, Figures 1, 2, 3, 4). Figures 1, 2, 3, 4 demonstrate that elderly patients with high comorbidity scores had higher 90‐day mortality rates than younger patients with low comorbidity scores.

## 4 DISCUSSION

The prognosis of resection of an adenocarcinoma in the head of the pancreas remains poor even after undergoing a pancreaticoduodenectomy with surgically negative margins.^8^, ^12^, ^42^, ^43^, ^44^, ^45^, ^46^ Previous studies found high perioperative morbidity and mortality rates for a pancreaticoduodenectomy, and the 30‐day mortality rate of a pancreaticoduodenectomy in a previous series was approximately 4%.^8^, ^12^, ^42^, ^43^, ^44^, ^45^, ^47^ The 30‐day mortality rates observed in previous studies are similar to our observations (ie, 4.12%). However, 90‐day postoperative mortality is a more‐legitimate measure of hepatopancreatobiliary surgical quality than is 30‐day mortality.^23^ Moreover, 30‐day mortality usually underestimates the risk of early death after major resections for malignancies.^10^, ^24^, ^25^ Therefore, we estimated the risk factors for 90‐day, not 30‐day, mortality after a pancreaticoduodenectomy in patients with PHA. The 90‐day mortality rate observed in our study was 8.39% and was double the 30‐day mortality after a pancreaticoduodenectomy for a PHA, which is compatible with results of Swanson et al^10^ In the modern era, a pancreaticoduodenectomy is also typically performed for head‐dominant chronic pancreatitis in patients who present with duodenal or biliary obstruction or an inflammatory mass in the head of the pancreas. In high‐volume centers, a pancreaticoduodenectomy results in pain relief in 70%‐89%, morbidity in 16%‐53%, and late mortality in <2% of patients with chronic pancreatitis.^48^, ^49^, ^50^ Compared to early mortality from a pancreaticoduodenectomy for a PHA, the early mortality rate is higher than late mortality from a pancreaticoduodenectomy for chronic pancreatitis. Therefore, a PHA may be considered per se a risk for greater morbidity. A pancreaticoduodenectomy for a PHA is also more complicated than other surgical procedures in the general surgery field due to a longer anesthesia time, more postoperative morbidities, and longer intensive care unit or hospital stay durations.^8^, ^12^, ^42^, ^43^, ^44^, ^45^, ^46^ Although we could not determine whether there were individuals who underwent a pancreaticoduodenectomy with venous resection in our cohort, we used AJCC clinical stages to adjust for the degree of difficulty in the pancreaticoduodenectomy. In addition, studies of easily assessed preoperative clinical factors for predicting 90‐day mortality after a pancreaticoduodenectomy for a PHA are scarce. There is no age‐comorbidity predicative scoring system for the high mortality rate of a pancreaticoduodenectomy in elderly patients with a PHA (Table 3). In our study, we used easily assessed preoperative clinical characteristics of patients with a PHA who received a pancreaticoduodenectomy to evaluate age comorbidity scores for predicting the early mortality rate.

In Table 1, the crude analysis of differences in demographics of patients with a PHA who received a pancreaticoduodenectomy showed high 90‐day mortality rates in elderly patients with a comorbidity, advanced AJCC stage, low hospital volume, and low income level. Unadjusted and risk‐adjusted 90‐day mortality rates were higher in patients of a geriatric age, with a higher clinical AJCC stage, with a lower income, and those who were operated on in a low‐volume hospital, which are compatible with findings of previous studies, although the time points of mortality assessment differed in previous studies.^10^, ^11^, ^19^, ^51^, ^52^, ^53^ Taiwan is defined as a developed country. In a comparison with data from other developed countries compatible with Taiwan, socioeconomic status influenced the likelihood of undergoing surgical treatment for pancreatic cancer in other developed countries.^54^, ^55^ Moreover, sample sizes for comorbidities such as hepatitis C, aortic aneurysm, and connective tissue diseases were smaller than those for other comorbidities; hence, the crude analysis showed no statistical difference with or without the comorbidity.

Development of bile duct obstruction may contribute to poor outcomes, including cholangitis, delay in treatment including chemotherapy or curative surgery, a decreased quality of life, and higher mortality.^56^ A pancreatic ductal adenocarcinoma has a dismal 5‐year survival rate of only 6%, and biliary obstruction was correlated with decreased survival times.^56^ As many as 70% of patients have some degree of biliary obstruction at the time of their initial diagnosis with pancreatic cancer.^57^ Occurrences of bile duct obstruction were 69.94% and 69.99% in the death and surviving groups, respectively. Bile duct obstruction was defined as an individual who underwent preoperative endoscopic retrograde cholangiography (ERCP), stents, percutaneous drainage, endoscopic ultrasound‐guided biliary drainage, or surgical bypass combined with ICD‐9 codes of jaundice. Bile duct obstruction had no statistical significance for 90‐day mortality in our cohort. ERCP is regularly performed in Taiwanese facilities before a pancreaticoduodenectomy.

A standard pancreaticoduodenectomy for removing lesions from within the head or uncinate process of the pancreas is also called the “Whipple procedure.”^58^ A standard pancreaticoduodenectomy involves a distal gastrectomy with removal of the pancreatic head, duodenum, first 15 cm of the jejunum, common bile duct, and gallbladder.^58^ A modification of the standard procedure, a pylorus‐preserving pancreaticoduodenectomy, preserves the gastric antrum, pylorus, and the proximal 2‐3 cm of the duodenum, which is anastomosed to the jejunum to restore gastrointestinal continuity.^59^ A pylorus‐preserving pancreaticoduodenectomy may decrease the incidence of postoperative dumping, marginal ulceration, and bile reflux gastritis that occur in many patients undergoing a partial gastrectomy, which is employed as part of the standard pancreatectomy technique.^59^ A pylorus‐preserving pancreaticoduodenectomy is not appropriate if the tumor involves the proximal duodenum, pylorus, or gastric antrum.^59^ In this circumstance, a standard pancreaticoduodenectomy operation should be done. The available data suggest that for suitable cases, perioperative morbidity and mortality, and long‐term survival are not affected by the use of pylorus‐preserving techniques.^60^, ^61^ The impact of a pylorus‐preserving approach on gastrointestinal function remains an open question.^60^, ^61^ To the present, there is no study of 90‐day mortality between standard and pylorus‐preserving pancreaticoduodenectomies. We designed the endpoint of 90‐day mortality based on curative‐intent and radical removal of all pancreatic cancer whether using a standard or pylorus‐preserving pancreaticoduodenectomy. Therefore, patients undergoing either a standard or pylorus‐preserving pancreaticoduodenectomy were enrolled in our cohort.

After conducting the multivariate Cox proportional hazard analysis of clinical factors for 90‐day mortality in patients with a PHA who received a pancreaticoduodenectomy, age subsets of 65‐74 and ≥75 years, COPD, sepsis, CKD, and dementia were determined as independent risk factors (Table 2). For patients with a PHA who received a pancreaticoduodenectomy, the aHR was higher for ≥75‐year‐old patients than for 65‐74‐year‐old patients. These findings are compatible with those of previous studies, which showed that old age is an independent risk factor for early mortality with a pancreaticoduodenectomy, but the cutoff points of age differed because time points of the mortality assessment which differed between our and previous studies.^11^, ^51^, ^62^

After adjustment, specific comorbidities such as CKD, sepsis, COPD, and dementia were determined to be independent risk factors for 90‐day mortality in patients with a PHA who received a pancreaticoduodenectomy (Table 2). Previous studies also indicated that COPD, CKD, and sepsis were independent risk factors for early mortality in patients who received a pancreaticoduodenectomy.^12^, ^52^, ^62^, ^63^ In our study, preoperative factors that were associated with 90‐day mortality in the multivariate analysis included an older age, COPD, sepsis, CKD, and dementia. The majority of these risk factors were associated with perioperative morbidity or mortality in previous studies on pancreatic resection results.^12^, ^52^, ^62^, ^63^ Overall morbidity after a pancreaticoduodenectomy associated with 90‐day mortality included preoperative factors that are considered risk factors for superficial and deep surgical site infection, pneumonia, unplanned intubation, renal insufficiency, and urinary tract infections.^12^, ^52^, ^63^, ^64^, ^65^, ^66^, ^67^, ^68^, ^69^, ^70^, ^71^, ^72^ COPD is a crucial patient‐related risk factor for postoperative pulmonary complications.^64^, ^65^, ^66^ Preoperative CKD is associated with an increased risk of complications and respiratory failure after pancreatic resection.^63^ Moreover, preoperative sepsis clearly alters innate and adaptive immune responses for a prolonged duration after clinical recovery, and such alterations include immune suppression, chronic inflammation, and bacterial persistence.^67^ Patients with a PHA and preoperative sepsis are susceptible to early mortality after a pancreaticoduodenectomy.^12^, ^52^ But sepsis might be related to cholangitis and directly associated with the pancreatic adenocarcinoma or a chronic preoperative comorbidity which could be difficult to clarify. We examined the covariate of sepsis, and outcomes showed preoperative sepsis to be a significant factor for 90‐day mortality after a pancreaticoduodenectomy as an easily assessed preoperative factor. Preoperative sepsis is an easily assessed preoperative factor; however, it might not be a chronic preoperative comorbidity. Similarly, previous studies also indicated that patients with preexisting dementia exhibit a high risk of early mortality after surgery, and their incidence of fatal complications was higher than that of surgical patients without dementia,^68^, ^69^, ^70^, ^71^ although surgery was not specifically a pancreaticoduodenectomy. Patients with preoperative dementia usually show low activity and poor self‐sufficiency, which increase postoperative complications, such as surgical site infection, urinary tract infections, and respiratory complications.^72^ Ours is the first study on patients with a PHA who received a pancreaticoduodenectomy, and the endpoint was 90‐day mortality after surgery. The results indicate that obvious and easily assessed independent risk factors for predicting 90‐day mortality in patients with a PHA receiving a pancreaticoduodenectomy are old age (≥65 years), COPD, CKD, sepsis, and dementia.

Other factors that were adjusted for in the multivariate analysis are listed in Table 2. These factors might be crucial, although they showed no statistical significance in the multivariate analysis. These factors might be bias‐selected by physicians’ initial decision on surgical‐intent for the patient with a PHA based on their experience, hospital volume level, and cancer clinical stage. The majority of patients with a PHA who received a pancreaticoduodenectomy were at clinical AJCC stages I (34.37%) and IIA (36.91%), while 24.77% and 3.86% of patients were at stages IIB and III (Table 1), respectively. Most surgeons consider stage III PHA to be an unresectable status without surgical‐intent treatment (Table 1). Surgeons at very‐low‐ and low‐volume hospitals might have selected AJCC clinical stage I PHA cases for a pancreaticoduodenectomy rather than advanced‐stage cases; hence, the clinical AJCC stage and hospital volume were statistically insignificant after adjustment (Table 2).

We used an age‐stratified Cox proportional hazard model to predict the 90‐day mortality risk associated with specific comorbidity scores in patients who received a pancreaticoduodenectomy (Table 3). In patients with the same comorbidity score (whether low, middle, or high) and a PHA who received a pancreaticoduodenectomy, elderly patients showed a higher 90‐day mortality rate than did younger patients (Tables 2 and 3, Figures 1, 2, 3, 4). Moreover, within the same age interval, patients with a PHA who received a pancreaticoduodenectomy with middle and high comorbidity scores showed higher 90‐day mortality rates than those with lower comorbidity scores (Tables 2 and 3, Figures 1, 2, 3, 4). These findings indicate that both age and specific comorbidity scores are independent risk factors for predicting 90‐day mortality in patients with a PHA who received a pancreaticoduodenectomy. A 90‐day mortality rate of 25% was observed in ≥75‐year‐old patients with high comorbidity scores (Table 3). Other alternative treatments, such as biliary or gastric bypass, endoscopic stents, targeted therapy, and chemotherapy or chemoradiation, might be considered, because the mean survival time of all of those treatments exceeded 90 days.^5^, ^73^, ^74^, ^75^ In Table 3, ≥75‐year‐old patients with a PHA and middle and high comorbidity scores showed extremely high 90‐day mortality rates. Whether the pancreaticoduodenectomy is effective in all patients with a PHA still remains unclear, especially for elderly patients with specific multiple comorbidities (Table 2). This aspect has been a challenge for physicians for a long time; hence, we attempted to establish predictive scores to enable further discussion between physicians and patients before a pancreaticoduodenectomy. The easily assessed preoperative predictive system described in our study could be a valuable and useful tool for both physicians and patients.

The strengths of our study are the inclusion of a very large cohort of patients with a PHA and the development of an easily assessed tool using preoperative characteristics such as age comorbidity scores to predict the 90‐day mortality for patients with a PHA who are to receive a pancreaticoduodenectomy (Table 3). In Tables 1 and 2, it is clear that surgeons selected patients with a PHA as affordable cases in their respective hospitals. However, although surgeons carefully selected patients, the 90‐day mortality was still high (8.39%) in Taiwan; a very high 90‐day mortality rate was noted in some patients with a PHA in this study, especially in elderly patients with a PHA and moderate or high comorbidity scores (Table 3). Our results obtained using age‐comorbidity predictive scores could be a valuable benchmark for selecting patients with a PHA who are to receive a pancreaticoduodenectomy with a high risk of 90‐day mortality. Further discussion about a pancreaticoduodenectomy between the physician and patient is worthwhile for PHA patients who are elderly with high comorbidities scores. This tool can be a reference for future clinical practice and was designed for selecting indicated patients in future trials.

This study has some limitations. First, as all of the patients with a PHA enrolled in this study were of Asian ethnicity, extrapolation of our findings to non‐Asian populations might not be entirely suitable. Moreover, as pancreatic cancer in Asians may be clinically similar to pancreatic cancer in Caucasians, the goals of future research on the disease might also be similar in these two ethnicities.^76^ The predictive scores still have clinical value as a pretreatment reference for a pancreaticoduodenectomy in patients with a PHA, whether patients are of Caucasian or Asian ethnicity. Second, diagnoses of all comorbidities were based on ICD‐9‐CM codes. However, to verify the accuracy of the diagnoses, the Taiwan Cancer Registry administration randomly reviews charts and interviews patients and ensures that hospitals with outlier chargers or practices are audited and subsequently heavily penalized if malpractice or discrepancies are identified. Furthermore, the quality and precision of ICD‐9‐CM codes in Taiwan have been verified and proven in previous studies.^35^, ^36^ Therefore, the conclusions of the study can be accepted as valid. Nevertheless, to obtain accurate information on population specificity and disease occurrence, a large‐scale randomized trial that carefully compares selected elderly patients with multiple comorbidities who have received suitable treatments is required. Finally, although informative, the TCRD lacks information such as that on dietary habits and the body‐mass index, both of which may be risk factors for 90‐day mortality in the context of a pancreaticoduodenectomy.

## 5 CONCLUSIONS

Elderly patients with a PHA and moderate or high comorbidity scores have a high risk of 90‐day mortality after a pancreaticoduodenectomy. Age‐comorbidity predictive scores for 90‐day mortality in patients with a PHA who receive a pancreaticoduodenectomy are easily assessed and are a valuable tool.

## CONFLICT OF INTEREST

The authors have no potential conflicts of interest to declare. The datasets supporting the study conclusions are included in the manuscript.

## AUTHOR CONTRIBUTIONS

*Conception and Design*: Ben‐Chang Shia, PhD; Lei Qin, Ph.D; Szu‐Yuan Wu, MD, MPH, PhD *Collection and Assembly of Data*: Ben‐Chang Shia, PhD; Lei Qin, PhD; Kuan‐Chou Lin, DDS, MS; Chih‐Yuan, Fang, DDS; Lo‐Lin Tsai, DDS, PhD *Data Analysis and Interpretation*: Ben‐Chang Shia, PhD; Lei Qin, PhD; Kuan‐Chou Lin, DDS, MS; Chih‐Yuan, Fang, DDS; Lo‐Lin Tsai, DDS, PhD *Administrative Support*: Szu‐Yuan Wu*. *Manuscript Writing*: All authors. *Final Approval of Manuscript*: All authors.

## ETHICS APPROVAL AND CONSENT

Our protocols were reviewed and approved by the Institutional Review Board of Taipei Medical University (TMU‐JIRB No. 201402018).

## CONSENT FOR PUBLICATION

Not applicable.

### AVAILABILITY OF DATA AND MATERIAL

The datasets supporting the study conclusions are included in this manuscript and its additional files.
